# 

**Published:** 1900-09-08

**Authors:** 


### NOTICE TO CORRESPONDENTS.

All MS., Letters, Books for Review, and other matters intended for the Editor should be addressed The Editor, "The Hospital," The Hospital Building, 28 & 29, Southampton Street, Strand, W.C.

The Editor cannot undertake to return rejected MS., even when accompanied by stamped directed envelope.

**Notice to Advertisers and Subscribers.**

All Advertisements, Orders for Copies of The Hospital, and Business Communications should be addressed to **THE MANAGER (Not to the Editor),** The Hospital, The Hospital Building, 28 & 29, Southampton Street, Strand, London, W.C.

The Cost of Subscription, post free, is as follows:—Per week, 2½d.; per quarter, 2s. 8d.; per half-year, 5s. 3d.; per annum, 10s. 6d. Foreign:—Per annum, 15s. 2d., payable, in all cases, in advance.

**NOTICE.—Vol. XXVII. of "The Hospital" (October, 1899, to April, 1900), in handsome cloth binding, is now on sale at the Office of this Journal, price 7s. 6d. Cases for binding the Numbers contained in this Volume 1s. 6d. Index to Vol. XXVII., 2½d., post free.**

**The Monthly Part for September is now ready. Price 6d., by post 8d.**

### The Student of Medicine.

### APPOINTMENTS.

E. A. Addison, M.R.C.S., L.R.C.P.Lond., appointed Medical Officer of the Workhouse and Coddenham District of the Bosmere and Claydon Union. W. J. A. Adye, M.R.C.S.Eng., appointed Medical Officer of Health for the Bradford-on-Avon Urban and Rural Districts. L. Bathurst, M.R.C.S., L.R.C.P.Lond., appointed Certifying Factory Surgeon for the Urban District of Ellesmere and the Civil Parishes of Ellesmere Rural, Welshampton, Cockshutt, and Hordley in Ellesmere Rural District. M. Bland, M.D.Glasg., appointed Medical Officer for the Guisborough District of the Guisborough Union. R. Burnet, M.B., Ch.B., B.Sc., appointed House Surgeon to the Chorley Dispensary

**THE STUDENT OF MEDICINE**—*continued.*

and Cottage Hospital. M. Caldwell, L.R.C.P., L.R.C.S.Edin., appointed Medical Officer for the Brotton District of the Guisborough Union. Dr. J. Clark, appointed Medical Officer for the Osmotherley District of the Northallerton Union. R. D. Clark, M.B., appointed Assistant Medical Officer to the Mill Road Infirmary of the West Derby Union. T. J. Connolly, M.B., appointed Medical Officer for the Kinvara District of the Gort Union. O. Eaton, M.R.C.S., L.R.C.P.Lond., appointed Medical Officer of Health to the Exmouth Urban District. J. H. Ferguson, F.R.C.S.I., D.P.H., appointed Medical Superintendent Officer of Health for the County Borough of Londonderry. J. Gray, M.B., C.M.Edin., appointed Medical Officer for the Middle Dispensary District of the Dundee Royal Infirmary. A. Greenwood, M.D., Ch.B., L.R.C.P., L.R.C.S., D.P.H., appointed Medical Officer of Health for the Borough of Crewe, and Medical Superintendent to the Crewe Isolation Hospitals. T. F. Hanley, L.R.C.P., L.R.C.S.Edin., appointed Medical Officer and Public Vaccinator for the Gillingham District of the Shaftesbury Union. W. C. Hayward, M.B., B.S.Durh., M.R.C.S., L.R.C.P., appointed Resident Medical Officer to the Kasr-el-Ainy Hospital, Cairo. A. G. R. Ingram, M.B.Aberd., appointed Certifying Factory Surgeon for the Helensburgh District. J. H. Jones, M.B., C.M.-Glasg., appointed Certifying Factory Surgeon for the Maesteg District of Glamorganshire. W. B. Kendall, L.R.C.P.Edin., M.R.C.S.-Eng., appointed Medical Officer for the Kingswear District of the Totnes Union. W. Kennedy, M.B.Lond., D.P.H.Camb., appointed Medical Officer for the Beaconsfield District of the Amersham Union. A. King, M.B., C.M.Edin., appointed Medical Officer of Health for the Watford Urban District. R. M. Littler, F.R.C.S.Eng., D.P.H.Vict., appointed Certifying Factory Surgeon for the Borough of Southport, Birkdale Urban District, and the Civil Parishes of Ainsdale and North Meols in Ormskirk Rural District. W. J. McCoy, M.R.C.S., L.R.C.P.Lond., appointed Second Assistant Medical Officer to the Poplar and Stepney Sick Asylum District. J. McKeith, M.B., C.M.Glasg., appointed Medical Officer to the Exeter Postal Staff. R. H. Manson, L.R.C.P., L.R.C.S.Edin., appointed Medical Officer of Health for the Darlington District of the Darlington Union. J. Panting, M.D.-Camb., L.R.C.S.Eng., appointed Certifying Factory Surgeon for the Watton District of Norfolk. H. Ramsden, M.D.Lond., appointed Medical Officer of Health for the Saddleworth Urban District. W. L. Rhys, L.R.C.P.Lond., M.R.C.S.Eng., appointed Certifying Factory Surgeon for the Urban Districts of Aberdare and Mountain Ash. W. H. Robinson, L.R.C.P.Lond., M.R.C.S.-Eng., appointed Medical Officer of Health for the Presall-with-Hackinsall Urban District. C. A. Spooner, L.R.C.P., L.R.C.S.-Edin., appointed Assistant Medical Officer of the Mile End Infirmary, Workhouse, and Schools. E. W. Sumpter, M.B., B.S.-Durh., appointed Medical Officer for the Cley District of the Erpingham Union. Dr. E. A. Sutherland, appointed Honorary Physician to the City Orthopædic Hospital. J. Thorney, L.R.C.P., L.R.C.S.Edin., appointed Medical Officer for the Skelton District of the Guisborough Union. C. Todd, M.D., D.P.H.Camb., appointed Assistant Bacteriologist to the Antitoxin Department of the Jenner Institute of Preventive Medicine in Sudbury, Harrow. J. P. Wagstaff, L.R.C.P.Lond., M.R.C.S.Eng., appointed Medical Officer for the No. 6 District of the Romford Union. E. C. Watts, M.D.Edin., appointed Medical Officer for the Guisborough Union Workhouse. W. R. E. Williams, M.B., Ch.B.Edin., appointed Assistant Medical Officer to the West Derby Union Infirmary. W. Wingrave, M.D., appointed Physician to the Central London Throat and Ear Hospital. W. Wynne, M.B., C.M.Edin., appointed Medical Officer of Health for the Rye Rural District.

St. Thomas's Hospital.—The following gentlemen have been selected as House Officers from Tuesday, September 4th, 1900. House Physicians: H. M. Harwood, M.A., M.B., B.C.Cantab.; R. B. Kinloch, L.R.C.P., M.R.C.S.; E. W. Hedley, M.A., M.B., B.C.Cantab. (extension); and F. C. Eve, B.A., M.B., B.C.Cantab. (extension). Assistant House Physicians: C. F. Selous, L.R.C.P., M.R.C.S.; and H. H. R. Clarke, L.R.C.P., M.R.C.S. House Surgeons: A. E. Martin, M.A., M.B., B.C.Cantab., L.R.C.P., M.R.C.S.; C. L. Hawkins, M.A., M.B., B.C.Cantab., L.R.C.P., M.R.C.S.; J. F. Cunningham, L.R.C.P., M.R.C.S.; and Y. Takaki, L.R.C.P., M.R.C.S. Assistant House Surgeons: T. H. Edwards, L.R.C.P., M.R.C.S.; B. S. Wills, L.R.C.P., M.R.C.S.; T. S. Taylor, M.A., M.B., B.C.Cantab.; and T. Burfield, M.A., M.B., B.C.Cantab., L.R.C.P., M.R.C.S. Obstetric House Physicians: (Senior) B. F. Howlett, L.R.C.P., M.R.C.S.; (Junior) H. R. Beale, L.R.C.P., M.R.C.S. Ophthalmic House Surgeon: (Senior) J. E. Kilvert, L.R.C.P., M.R.C.S. (extension). Clinical Assistant in the Special Department for Diseases of the Skin: C. A. R. Nitch, L.R.C.P., M.R.C.S. (extension). Clinical Assistant in the Special Department for Diseases of the Ear: Z. Mennell, L.R.C.P., M.R.C.S. Clinical Assistant in the Electrical Department: L. S. Dudgeon, L.R.C.P., M.R.C.S.

Royal 8vo., cloth gilt, illustrated by a series of Original Photo-micrographs and many Illustrations in the Text, 7s. 6d. net.

**THE MENOPAUSE AND ITS DISORDERS.**

**With Chapters on Menstruation.**

By A. D. LEITH NAPIER, M.D., M.R.C.P., late Editor of the *British Gynæcological Journal.*

"Of great practical use to the general practitioner as well as to those more specially engaged in gynæcological work."—*The Hospital.*

London: The Scientific Press, Ltd., 28 & 29, Southampton Street, Strand, W.C.

### NOW READY,

A REPRINT OF

### A NURSING HANDBOOK

TOGETHER WITH S. MAW, SON, AND THOMPSON'S

### COMPLETE CATALOGUE OF NURSING REQUISITES.

The above work is now republished at a cost of 1s. per copy. Messrs. S. M., S., and T. have been compelled to make this charge on account of the extreme popularity of the work and the great expense incurred in its production.

***Post Free on receipt of 1s.***

Nurses' Bags.

### S. MAW, SON, and THOMPSON,

7 to 12, ALDERSGATE STREET, LONDON, E.C.

